# The effects of workplace respect and violence on nurses’ job satisfaction in Ghana: a cross-sectional survey

**DOI:** 10.1186/s12960-018-0269-9

**Published:** 2018-01-15

**Authors:** Isaac Mensah Boafo

**Affiliations:** Department of Sociology, University of Ghana, Legon, Accra, Ghana

**Keywords:** Job satisfaction, Violence, Verbal abuse, Respect, Interactional justice, Ghana

## Abstract

**Background:**

Studies have established the negative effects of workplace disrespect and violence on the personal and professional well-being of nurses. In spite of this, only a few have directly investigated the effects of these issues on nurses’ job satisfaction. In Africa, research on nurses’ job satisfaction continues to focus largely on economic factors. The aim of this paper was, therefore, to investigate the impact of the non-economic factors of workplace violence and respect on the job satisfaction levels of nurses in Ghana.

**Methods:**

The study employed a cross-sectional questionnaire survey. It involved 592 qualified practising nurses working in public hospitals in Ghana. Data were collected between September 2013 and April 2014.

**Results:**

The results showed that, overall, nurses were neither satisfied nor dissatisfied with their jobs (*M =* 3.19, SD = .54). More than half (52.7%) of the participants had been abused verbally, and 12% had been sexually harassed in the 12 months prior to the study. The majority of nurses, however, believed they were respected at the workplace (*M =* 3.77, SD = .70, Mode = 4). Multiple regression analyses showed that verbal abuse and perceived respect were statistically significant predictors of nurses’ job satisfaction. Nurses who experienced verbal abuse and low level of respect were more likely to report low job satisfaction scores.

**Conclusion:**

It is concluded that non-financial strategies such as safe work environments which are devoid of workplace violence may enhance nurses’ job satisfaction levels. A policy of “zero tolerance” for violence and low tolerance for disrespect could be put in place to protect nurses and healthcare professionals in general.

## Background

Nurses’ productivity and the quality of care they provide depend largely on the availability of adequate nursing staff [1], thus making the shortage and high turnover rates of nurses a topical issue globally [2]. The nurse-patient ratio is perceived to be inadequate in many countries, with sub-Saharan Africa being the worst affected region [3]. Job satisfaction contributes to this global shortage as it is often linked to nurses exiting the profession [4–7].

Studies conducted on the job satisfaction levels of hospital-based nurses show that over a quarter are dissatisfied [8–11]. Sources of dissatisfaction include collegial relationships and leadership [12–14], education and promotion prospects [4, 15], autonomy [12, 13, 16] and pay [11, 13, 17, 18]. Other factors such as the type of hospital (e.g. teaching and non-teaching), the location of hospital (rural or urban), the unit/department where one works, and the work environment have also been shown to impact nurses’ job satisfaction [19–22].

Nurses’ job satisfaction is, however, not uniformly influenced by these factors. Whereas some are most dissatisfied with pay and promotion prospects [15], others are reported to be most dissatisfied with childcare facilities, compensation for working weekends and control over working conditions [23]. Studies, particularly from low- and middle-income countries, suggest that pay levels exert great influence on the satisfaction levels of nurses. For instance, in a survey of nurses from five hospitals in Addis Ababa, Ethiopia, salary was found to be the key and pronounced factor influencing job satisfaction with a correlation coefficient of 0.74 [24]. Mohite et al. [25] reported in their Indian study that although nurses reported high scores on satisfaction with their level of achievement and utilization of their abilities, they expressed dissatisfaction with the level of compensation, which statistically reduced their level of overall satisfaction.

Job satisfaction levels of nurses in different geographical and socio-cultural settings need to be assessed to determine which factors impact on them the most. This may assist healthcare managers and policy makers to prioritize the issues which must be addressed to improve the satisfaction levels of nurses, and consequently healthcare delivery. This is particularly important for less-developed economies such as Ghana, where resource constraints are very pronounced. Arguably, non-financial factors impacting negatively on nurses’ job satisfaction when addressed may have a positive ripple effect on overall job satisfaction.

Workplace violence, which refers to “incidents where staff are abused, threatened or assaulted in circumstances related to their work, including commuting to and from work, involving an explicit or implicit challenge to their safety, well-being or health” [26] has been found to be a major problem for nurses globally [27–33]. Available evidence suggests that rates of workplace violence against nurses may range from 9% to as high as 89% [34, 35]. Both physical and non-physical forms of workplace violence have detrimental effects on the physical and psychological well-being of nurses, and consequently quality of care they provide, and the rate at which nurses change jobs or leave the profession [27, 32, 36–39]. Such effects range from physical injuries to having symptoms related to post-traumatic stress disorder [27, 32, 34].

Closely related to the issue of workplace violence is that of respect given to nurses at the workplace. Respect is a moral principle that involves valuing the dignity and worth of another person [40, 41]. The notion of respect is present in all cultures, but the behaviours that express respect differ across cultures [42]. Respect influences quality of care [43], recruitment and retention as it impinges on job satisfaction of nurses [42]. Moreover, the global shortage of nurses dictates that strategies put in place to attract individuals into the profession extend beyond financial incentives to include respecting nurses within the healthcare setting [44].

The concept of respect is usually found in research on organizational justice [45]. It falls under the category of interactional justice, which refers to the “perceptions of the quality of interaction among individuals...” [44]. Evidence from fields outside nursing suggests that in order to keep employees satisfied and committed to an organisation, they need to be treated fairly and respectfully [46, 47]. In a study of sports federation workers for instance, it was found that interactional justice correlated positively with job satisfaction and was negatively related to turnover intentions [48].

Although studies often allude to lack of respect as a concern for nurses, few studies on respect exist in the nursing literature [44]. Cotter et al. [49] noted that apart from those aligned with nursing, the perception of many people is to consider nursing as inferior. Other authors support this position by contending that nurses often do not get the respect they deserve from other health professionals, particularly physicians [50–53]. Many nurses also experience lack of respect from patients and their relatives [54].

Like workplace violence, disrespect for nurses can have personal and professional impacts. Persistent experience of disrespect may lead to fear, anger, humiliation and lowered job satisfaction, reduced work performance, turnover intentions and actual turnover, insomnia and hypertension [43, 55]. Expression of disrespect especially by physicians may also strain inter-professional communication and collaboration [42, 55]. Consequently, Middleton [50] contended that “when nurses have respect, care is better”.

In spite of evidence pointing to the association between respect and occupational outcomes, there is a dearth of studies directly investigating the association between nurses’ perception of the respect they receive and their job satisfaction. This data scarcity also holds true for the association between workplace violence and nurses’ job satisfaction, particularly in Africa [56]. In Ghana, studies on nurses’ job satisfaction have largely focused on resource-related predictors such as salaries, opportunities for further education and promotion, poor physical environments and lack of materials and basic equipment [9, 57]. It is also remarkable that most studies on interactional justice focus on the relationships among members of an organisation to the exclusion of relationships among employees and clients or outsiders [42, 46, 47].

This paper is, therefore, concerned with investigating the impact of perceived respect and workplace violence on Ghanaian nurses’ job satisfaction. The current paper makes a significant contribution to the body of knowledge by providing empirical evidence to the effect that job satisfaction of nurses in Africa is not only influenced by economic factors but also other non-economic factors such as violence and lack of respect at the workplace. Indeed, the current study is in line with the principles of the WHO’s Global Strategy on Human Resource for Health: Workforce 2030 [58]. One of the principles of this strategy is to ensure the personal, employment and professional rights of all health workers**,** including safe and decent working environments and freedom from all kinds of discrimination, coercion and violence.

The current paper provides answers to the following research questions:

1. Do Ghanaian nurses believe they are respected at their workplaces?
2. What is the level of job satisfaction among Ghanaian nurses?
3. What is the relationship between workplace respect and violence and nurses’ job satisfaction?

## Methods

This paper is a part of a larger study of workplace experiences of Ghanaian nurses. A cross-sectional descriptive questionnaire survey was conducted between September 2013 and April 2014 in 12 hospitals in Ghana comprising of two teaching hospitals, five regional hospitals and five district hospitals.

### Sampling—hospitals

Five out of the ten regions of the country were purposively selected for the study. These were Northern, Ashanti, Greater Accra, Eastern and Volta. The reason for selecting these regions was to ensure that all three major ecological zones, namely, the coastal, forest and savannah zones, were represented. It also ensured that the various social, cultural, economic and demographic characteristics of the entire country were captured.

Nine of the ten regions had a regional hospital. The regional hospitals in the selected regions were automatically selected for the study. In the Ashanti Region where according to the Ghana Health Service no hospital is designated as a regional hospital [59], the Suntreso Government Hospital which is located in the Kumasi metropolis was chosen to represent a regional hospital due to its location and the diversity of the people it serves. Two of the three teaching hospitals in the country were selected for the study. The Korle Bu and Tamale teaching hospitals located in the Greater Accra and Northern Regions respectively were selected to ensure the sample was representative of the northern/southern divide of the country. Finally, one district hospital was randomly selected from each of the five selected regions. Data on the districts in Ghana were obtained from the Ghana Statistical Service [54]. The selection of district, regional and teaching hospitals was to ensure that all the three levels (primary, secondary and tertiary) of healthcare delivery in Ghana were captured. The location, size and the type of cases handled by these hospitals may have implications for workplace violence, disrespect and nurses’ job satisfaction.

### Sampling—participants

Data were collected using a multi-stage sampling technique. In the first stage, Korle Bu and Tamale teaching hospitals were purposively selected to ensure that both northern and southern sectors of the country were represented. The second stage involved selecting five regional hospitals from five randomly selected regions. And in the third stage, one district hospital was randomly selected from each of the randomly selected regions. In each of the selected hospital, nurses were then selected from the various units/departments using a simple random sampling technique. Data collection took place between 12:00 and 21:00 to ensure that nurses working all shifts had equal opportunity for participation. The data were collected using printed (hardcopy) questionnaires. To participate in the study, one had to be a professional nurse and should have practiced for at least 12 months. A total of 1021 professional nurses were invited to take part in the survey, of which 685 accepted to participate and 592 returned questionnaires were valid for statistical analyses, giving a net response rate of 58%. Data were collected by the researcher and four trained research assistants. No incentives were provided for participation.

### Instrument

A self-report questionnaire was utilized for data collection. Five nurses reviewed the questionnaire for face validity, clarity and sensitivity of items. Three of the nurses were from teaching hospitals, and two from district hospitals. Based on their feedback, necessary adjustments were made before the start of the study. The final questionnaire contained items on workplace violence, perceived respect, job satisfaction and socio-demographic variables.

The study utilized the Health Sector Workplace Violence Questionnaire jointly developed by the International Labour Organization, International Council of Nurses, World Health Organization and Public Services International [26]. This questionnaire was used to ensure that the incidence of violence reported in this study could be compared with other studies which have utilized the questionnaire. The three forms of violence—physical, verbal and sexual—were measured using single-item scales and for that matter it was not possible to determine their reliabilities using Cronbach’s alpha [60]. In view of this, prior to the study, these items were tested on 20 nurses who were not part of the study on two occasions with a 2-week interval. The test-retest correlation coefficients for sexual abuse, verbal abuse and physical violence were 1.00, 1.00 and .90 respectively.

The “perceived respect” variable was informed by Siegrist’s [61] Esteem Scale. The original scale contains three items measuring nurses’ perception of the respect they receive from managers and peers. The scale was modified to include respect from patients, relatives of patients, doctors and other hospital staff. The scale in the present study thus contained seven items rated on a 5-point Likert scale (1 = strongly disagree; 5 = strongly agree). The internal consistency of the scale as measured by Cronbach’s alpha was 0.86. The scale was constructed by computing the averaged sum score.

The job satisfaction scale was informed by the Measure of Job Satisfaction scale (MJS) [62]. The scale used in the current study was constituted by 12 items. The items on the scale covered different aspects of the job including work organisation, pay and promotion prospects, education and training prospects, and professional relationships.

The items were measured on a 5-point Likert type scale with responses ranging from 1 (strongly dissatisfied) to 5 (strongly satisfied). The overall scale had adequate internal consistency with a Cronbach’s alpha of .77. The overall satisfaction scale was constructed by computing the mean sum score for the items. The methods used in this study have also been published elsewhere [63].

## Results

### Demographic and workplace characteristics

A relative majority (42.1%) of the participants worked in the Greater Accra Region. Over one fifth (22.8%) of the participants were nurses in the Eastern Region. More than a third (38.7%) of the participants worked in regional hospitals, and a third (32.9%) worked in teaching hospitals.

Approximately 20% of the participants were males and 80% were females. Their ages ranged from 21 to 60 years with a mean age of 31.76 years (SD = 9.69). More than half of the sample (61.3%) had Diploma qualifications (see Table 1). In terms of position/rank, 53% were Staff Nurses, 22.7% were Senior Staff Nurses and 4.8% were Principal Nursing Officers (see Table 2). Participants had been in the nursing profession for between 1 and 40 years (*M =* 7.38, SD = 9.53); 17% worked in the outpatient department (OPD), and 35.5% worked at medical and surgical units. Demographic and workplace characteristics of the participants are displayed in Table 1.

**Table 1:** Socio-demographic characteristics of participants (*N* = 592)

Item description	*n* (%)
Region	
Greater Accra	249 (42.1)
Eastern	130 (22.0)
Ashanti	76 (12.8)
Volta	51 (8.6)
Northern	86 (14.5)
Gender	
Female	469 (79.2)
Male	123 (20.8)
Age group	
21–30	398 (67.7)
31–40	98 (16.7)
41–50	37 (6.3)
51–60	55 (9.4)
Marital status	
Single	310 (52.5)
Married	280 (47.5)
Educational attainment	
Certificate	79 (13.4)
Diploma	363 (61.7)
Bachelor’s degree or higher	146 (24.8)
Age (min = 21, max = 60, mean = 31.76, SD = 9.69)	
Hospital type	
District hospital	168 (28.4)
Regional hospital	229 (38.7)
Teaching hospital	195 (32.9)
Units/department	
Critical care	119 (20.2)
Outpatient department	100 (17.0)
Medical-surgical unit	209 (35.5)
Special units	160 (27.2)
Position/grade	
Staff Nurse	308 (53.0)
Snr. Staff Nurse	132 (22.7)
Nursing Officer	72 (12.4)
Snr Nursing Officer	42 (7.2)
Principal Nursing Officer	28 (4.8)

Source: Field Survey 2013–2014

**Table 2:** Incidence of violence

Item descriptions	*n* (*n*%)
Have you been verbally abused in your work place in the past 12 months?	
Yes	312 (52.7)
No	280 (47.3)
Have you been sexually harassed in your workplace in the past 12 months?	
Yes	72 (12.2)
No	520 (87.8)
Have you been physically abused in your workplace in the past 12 months?	
Yes	53 (9.0)
No	539 (91.0)
How concerned are you about violence (min = 1, max = 5, mean = 3.05, SD = 1.51)	
Number of years in nursing (min = 1, max = 40, mean = 7.38, SD = 9.53)	

Source: Field Survey 2013–2014

### Perceived respect

The mean scores of the major sub-groups on the respect items are presented in Table 3. Males reported higher mean scores on almost all the items making up the scale. However, the differences in the means were very marginal (< .5). Small differences were also found among nurses working in the various regions of the country. Greater Accra Region recorded the lowest mean scores with regard to respect from patients (*M =* 3.45) and relatives of patients (3.19). Although marginal, this systematic difference may be explained by inadequate staffing and overcrowding in public hospitals within the region, which may in turn lead to dissatisfaction among consumers of health care. With regard to position, senior nurses scored higher on all the items making up the respect scale compared to junior ones. Junior nurses were more likely to be young and single. Of those who were single, 85.7% were junior nurses and 14.3% were senior nurses (*N* = 580, df = 1, *χ*^2^ = 36.94, *p* = .000).

**Table 3:** Nurses’ experience of respect

I receive respect I deserve from	Patients	Relatives of patients	Colleagues	Supervisors	Doctors	Other staff
Females						
Mean *N* SD	3.51 467 1.07	3.32 467 1.12	3.96 467 .86	3.85 467 .89	3.73 467 .90	3.90 467 .80
Males						
Mean *N* SD	3.80 122 .99	3.64 122 1.00	4.09 122 .85	3.96 122 .87	3.70 121 .100	3.98 122 .87
Northern Region:						
Mean *N* SD	3.72 86 .98	3.58 86 .100	4.06 86 .74	4.03 86 .77	3.63 86 1.00	3.91 86 1.01
Volta Region:						
Mean *N* SD	3.61 51 1.15	3.49 51 1.16	3.76 51 .97	3.71 51 1.04	3.86 50 1.03	3.88 51 .86
Ashanti Region:						
Mean *N* SD	3.72 75 1.01	3.68 75 1.02	4.13 75 .88	3.88 75 .96	3.71 75 1.05	3.92 75 .93
Eastern Region:						
Mean *N* SD	3.60 130 .96	3.43 130 .95	4.05 130 .70	3.94 130 .72	3.68 130 .74	3.95 130 .70
Greater Accra:						
Mean *N* SD	3.45 247 1.20	3.19 247 1.12	3.94 247 .93	3.81 247 .94	3.75 247 .91	3.92 247 .82
Junior Nurses:						
Mean *N* SD	3.46 438 1.08	3.32 438 1.11	3.97 438 .89	3.83 438 .89	3.67 437 .91	3.88 438 .83
Senior Nurses:						
Mean *N* SD	3.85 141 .95	3.56 141 1.08	4.06 141 .77	4.01 141 .83	3.87 141 .93	4.04 141 .76
Total Sample:						
Mean *N* SD	3.57 589 1.06	3.39 589 1.10	3.99 589 .86	3.87 589 .89	3.72 589 .92	3.92 589 .89

Source: Field survey 2013–2014

*Other staff* laboratory technicians and other allied health professionals

Overall, the results as presented in Table 3 show similar average ratings for all items on the respect scale (3 < M < 4). The standard deviations of the various items on the scale were small, suggesting that the means were highly representative of the scores on each item. In fact the modal mark for all the items on the respect scale was four. This means that a relative majority of nurses “agreed” to statements that they were respected at the hospital.

The mean score of the respect scale was 3.77 (SD = .70, Mode = 4) with possible scores ranging from 1 to 5. These results, therefore, suggest that, in general, nurses believed they were respected by staff and consumers of healthcare services since the mean score was above the mid-point of the scale and the modal score was four.

Independent samples *t*-test conducted to compare the scores for male and female nurses on the respect scale revealed a statistically significant difference, *t*(586) = − 3.04, *p* = .03, two-tailed, with male nurses (*M =* 3.87, SD = 0.70) scoring higher than female nurses (*M =* 3.71, SD = 0.70). Further analyses of the level of perceived respect from the various actors in the hospitals also suggested that male nurses received a statistically significantly higher level of respect from patients’ relatives (*M =* 3.64, SD = 1.0) compared to female nurses (*M =* 3.32, SD = 1.12), *t*(208.69) = − 3.06, *p* < .01. Similarly, there was a statistically significant difference in the level of respect received from patients, *t*(201.96) = − 2.79, *p* = .01, by male (*M =* 3.80, SD = .99) and female (*M =* 3.51, SD = 1.07) nurses. Usually, these findings would mean that male nurses believed they were given greater level of respect than their female counterparts at the hospital. However, the differences in the means are almost negligible. The ratings of both male and female nurses fell within the same qualitative category on the scale. Moreover, the same modal score (four) was recorded for both male and female nurses on the perceived respect scale, and on the items making up the scale. This means that in real-life situations, there may be no gender disparities in nurses’ perceptions/experiences of respect at the workplace. No statistically significant difference was found between male and female nurses with regard to the level of respect received from doctors.

A comparison of the scores of junior and senior nurses on the perceived respect scale showed a statistically significant difference in the means, *t*(576) = − 3.04, *p* < 0.01 (two-tailed) with junior nurses (*M =* 3.70, SD = .72) rating the respect they receive lower than senior nurses (*M =* 3.90, SD = .62). The individual items on the respect scale revealed a statistically significant difference between senior (*M =* 3.56, SD = 1.08) and junior (*M =* 3.32, SD = 1.08) nurses with regard to their perception of the level of respect received from relatives of patients, *t*(577) = − 2.23, *p* < .05. Senior nurses perceived a statistically significantly higher level of respect from doctors (*M =* 3.87, SD = .93) compared to junior nurses (*M =* 3.67, SD = .91), *t*(576) = − 2.17, p < .05.

### Workplace violence

The results indicated that 53 (9%) experienced physical violence; 312 (52.7%) had suffered verbal abuse; and 72 (12.2%) reported that they have been sexually harassed in their workplaces in the 12 months prior to the study. Although the mean score for nurses’ concern over workplace violence suggests that nurses were moderately concerned about workplace violence, the mode revealed that many nurses were extremely concerned about workplace violence (min =1, max = 5, mean = 3.05, SD = 1.51, mode = 5). Being concerned may mean that nurses did not feel safe in their workplaces as far violence is concerned. It is an admission that they are at risk of violence. These results are worrying particularly considering the fact that creating a violence-free atmosphere at the workplace is one of the principles underlying WHO’s Global Strategy on Human Resource for Health [58]. Table 2 provides data on the incidence of workplace violence among the sample. A more detailed analysis of the incidence of workplace violence among the sample has also been reported elsewhere [32].

### Job satisfaction

The overall job satisfaction scale had a mean score of 3.19 (SD = 0.54), with higher scores indicating greater satisfaction levels. This result means that, in general, Ghanaian nurses were neither satisfied nor dissatisfied.

One-way analysis of variance (ANOVA) suggested that, statistically, nurses working in different types of hospitals (district, regional and teaching hospitals) did not differ in a significant way with regard to their overall job satisfaction. Independent samples *t*-test comparing the scores on the overall scale revealed a statistically significant difference in the scores of female (*M =* 3.17, SD = 0.53) and male (*M =* 3.30, SD = 0.55) nurses; *t*(581) = − 2.36, *p* = .02. The means, however, fell within the same qualitative response category (neither satisfied nor dissatisfied) on the scale. The statistically significant difference could, therefore, be the result of the large sample size employed. In real-life situations, there may be no difference between the job satisfaction levels of males and females in Ghana.

With regard to position, the results of an independent samples *t*-test indicated that junior and senior nurses had similar mean scores on the overall job satisfaction scale.

To fulfill the main objective of this paper, the relationship between workplace respect, violence and job satisfaction was explored using Pearson’s correlation. Statistically significant relationships were found between workplace physical violence, verbal violence, perceived respect and job satisfaction. Negative associations were found between workplace violence and job satisfaction. On the other hand, a positive association was found between perceived respect and job satisfaction. Results of the bivariate analyses as presented in Table 4 suggest that workplace verbal abuse explains approximately 5% of the variance in nurses’ job satisfaction, and perceived respect explains 14.4% of the variance in nurses’ job satisfaction. These results suggest that it is possible to enhance nurses’ job satisfaction through non-economic (financial) means.

**Table 4:** Correlational analyses—workplace violence, perceived respect and job satisfaction

		Physical violence	Sexual harassment	Verbal abuse	Respect received	Job satisfaction
Physical violence	Pearson correlation	1	.03	.20**	− .05	− .09*
	Sig (two-tailed)		.46	.000	.26	.03
	*N*	592	591	590	588	581
Sexual harassment	Pearson correlation	.03	1	.28**	− .09*	− .07
	Sig (two-tailed)	.461		.000	.03	.09
	*N*	591	591	589	587	580
Verbal abuse	Pearson correlation	.202**	.28**	1	− .19**	− .22**
	Sig (two-tailed)	.000	.000		.000	.000
	*N*	590	589	590	587	579
Perceived respect	Pearson correlation	− .046	− .09*	− .19**	1	.38**
	Sig (two-tailed)	.264	.03	.000		.000
	*N*	588	587	587	588	578
Job satisfaction	Pearson correlation	− .091*	− .07	− .22**	.38**	1
	Sig (two-tailed)	.03	.09	.000	.000	
	*N*	581	580	579	578	581

**Correlation is significant at the .01 level (two-tailed)

*Correlation is significant at the .05 level (two-tailed)

### Predicting job satisfaction

Standard multiple regression analysis was used to assess the ability of selected socio-demographic and workplace characteristics to predict nurses’ job satisfaction. The analyses were checked to ensure that the assumptions of normality, linearity, multicollinearity, and homoscedasticity were not violated [64]. The prediction model for nurses’ job satisfaction suggested that perceived respect (*t* = 9.12, *p* < 01), experience of workplace verbal abuse (*t* = − 3.22, *p* < .01) and the rank of a nurse (*t* = − 3.21, *p* < .01) were the significant predictors of nurses’ job satisfaction. The *Beta* values as presented in Table 4 suggest that perceived respect makes the strongest unique contribution towards explaining the variability in nurses’ job satisfaction. The overall model accounted for 18% of the variance in nurses’ job satisfaction (see Table 5).

**Table 5:** Predicting Ghanaian nurses’ job satisfaction

Variable	*B*	SE	Beta	*t*	*p* value	95% CI for *B*
Constant	2.27	.12	–	19.01	.00	2.04 to 2.51
Gender						
Female	Reference	–	–	–	–	–
Male	.05	.05	.04	1.01	.32	− .05 to .15
Position						
Junior Nurse	Reference	–	–	–	–	–
Senior Nurse	− .16	.05	− .13	− 3.21	.00	− .25 to − .06
Verbally abused						
No	Reference	–	–	–	–	–
Yes	− .14	.05	− .13	− 3.22	.00	− .23 to − .06
Physically abused						
No	Reference	–	–	–	–	–
Yes	− .07	.07	− .04	− .94	.35	− .22 to .08
Sexually harassed						
No	Reference	–	–	–	–	–
Yes	.01	.07	.00	.11	.92	−.12 to .14
Perceived respect	.28	.03	.36	9.12	.00	.22 to .33
*R*^2^ = .179	*F* (df = 6, *N* = 566) = 20.26, *p* = .000					

Source: Field Survey 2013–2014

## Discussion

The mean score for overall job satisfaction among Ghanaian nurses was found to be 3.19 (range 1–5). Nurses were, therefore, on average neither satisfied nor dissatisfied with their jobs. This finding of neutrality regarding satisfaction is consistent with other international studies on nurses from countries such as Israel and Jordan [17, 65].

One major finding of the current study was the fact that workplace verbal violence and perceived respect were statistically significant predictors of nurses’ job satisfaction. Nurses who were exposed to verbal abuse were less satisfied with their job. This finding is consistent with the findings of other studies which have reported a negative association between workplace violence and nurses’ job satisfaction [66–69]. This finding can also be related to other studies [70, 71], which have reported associations between verbal abuse and turnover rates among nurses. If job satisfaction is about the way people think and feel about their work, then how individuals think and feel about certain experiences associated with their jobs such as verbal violence and interactional justice, can impact their satisfaction levels.

On the issue of perceived respect, it was found that with the exception of respect from medical doctors, male nurses perceived their level of respect to be higher than female nurses. Although the mean difference was marginal, this could be as a result of the fact that within the Ghanaian context, male nurses are usually mistaken for medical doctors by patients and their relatives. It could also be the result of the age-old tradition where males are seen as authority figures and revered more than women. Another interesting finding in relation to perceived respect was that junior nurses rated their respect lower than senior nurses. In the Ghanaian setting, older people are given higher respect than younger persons. Similarly, people who are married are seen as being more responsible and are, therefore, accorded higher respect than unmarried individuals [72]. This could explain why junior nurses reported lower respect than senior nurses, since senior nurses are more likely to be older and married. Another plausible explanation could be that senior nurses have more experience and are able to relate better with patients and their relatives.

The current study also established a positive association between the level of perceived respect and nurses’ job satisfaction. Nurses may derive satisfaction from their job if they are respected and valued in the hospital and by the general society. Indeed, it is logical and rational for people to be less satisfied with aspects of their job such as pay if the job is fraught with stress induced by violence and disrespect. If nurses in developing countries such as Ghana work in environments where their personal and professional rights are respected, including working in safe environments and freedom from violence and disrespect, they may be more likely to be less dissatisfied with their pay and other financial rewards.

Although a number of studies have tried to measure the job satisfaction levels of nurses in Ghana, all these studies have concentrated on job components such as salary and opportunities for professional development [9, 57]. None of these studies have assessed the impact of workplace violence on nurses’ job satisfaction. The findings of the current paper have showed that for Ghanaian nurses, although pay and promotion were important areas of dissatisfaction, other factors such as workplace verbal violence, perceived respect and staffing adequacy were significant determinants of nurses’ job satisfaction. The results of the study reported in the current paper would therefore be of great importance to policy makers and hospital administrators who seek to improve the job satisfaction levels of nurses. As per the results, the concentration must not always be on how to provide financial incentives for nurses. Efforts must also be put into creating a safe and civilised work environment for nurses. This, indeed, is in line with the WHO’s Global Strategy for Health Workforce [58].

### Limitations

This study involved nurses working in public hospitals in Ghana. Its findings may not be applicable to nurses working in the private sector. The cross-sectional nature of the study also implies that causal inferences cannot be drawn from it. The current study measured nurses’ levels of job satisfaction and their perceptions of respect accorded them at the workplace. In terms of respect, they believed they were generally respected, and with regard to satisfaction, they were generally neutral. However, the study was unable to establish how these compare with other healthcare professionals. For instance, questions such as are nurses more satisfied than medical doctors or hospital-based pharmacists? Do nurses report greater levels of respect than medical doctors or hospital-based pharmacists? These questions could not be answered by the current study. The limitations identified, however, provide avenues for future research.

## Conclusion

The current paper established that verbal abuse, respect and staffing adequacy influenced nurses’ job satisfaction levels. In a resource constrained country such as Ghana, it may not always be possible to increase job satisfaction levels through financial incentives. However, policies aimed at reducing workplace violence, ensuring low tolerance for workplace disrespect and increasing staffing levels of nurses may go a long way to improve the satisfaction levels of nurses. Indeed, the Global Strategy on Human Resources for Health [58] underscores the importance of creating a safe work environment devoid of workplace violence in improving the quality of health workforce and health.

## References

[1] Hassmiller SB, Cozine M (2006). Addressing the nurse shortage to improve the quality of patient care. Health Aff.

[2] Oulton JA (2006). The global nursing shortage: an overview of issues and actions. Policy, Politics, & Nursing Practice.

[3] Buchan J, Aiken L (2008). Solving nursing shortages: a common priority. J Clin Nurs.

[4] Lu H (2012). Job satisfaction among hospital nurses revisited: a systematic review. Int J Nurs Stud.

[5] Samir N (2012). Nurses’ attitudes and reactions to workplace violence in obstetrics and gynaecology departments in Cairo hospitals. East Mediterr Health J.

[6] De Gieter S, Hofmans J, Pepermans R (2011). Revisiting the impact of job satisfaction and organizational commitment on nurse turnover intention: an individual differences analysis. Int J Nurs Stud.

[7] Bonenberger M (2014). The effects of health worker motivation and job satisfaction on turnover intention in Ghana: a cross-sectional study. Hum Resour Health.

[8] Hayes B, Bonner A, Douglas C (2013). The levels of job satisfaction, stress and burnout in Australian and New Zealand haemodialysis nurses.

[9] Anarfi J, Quartey P, Agyei J (2010). Key determinants of migration among health professionals in Ghana.

[10] Lu H, While AE, Barriball KL (2007). Job satisfaction and its related factors: a questionnaire survey of hospital nurses in mainland China. Int J Nurs Stud.

[11] Adams A, Bond S (2000). Hospital nurses’ job satisfaction, individual and organizational characteristics. J Adv Nurs.

[12] Cummings GG (2008). The relationship between nursing leadership and nurses’ job satisfaction in Canadian oncology work environments. J Nurs Manag.

[13] Bjørk IT (2007). Job satisfaction in a Norwegian population of nurses: a questionnaire survey. Int J Nurs Stud.

[14] Williams D (2006). Looking for a few men.

[15] White MJ, White GB (2006). Implicit and explicit occupational gender stereotypes. Sex Roles.

[16] Zangaro GA, Soeken KL (2007). A meta-analysis of studies of nurses’ job satisfaction. Research in nursing & health.

[17] Tollison AC. Stereotype threat in male nurse-patient interactions. The University of Texas at Austin: Texas. 2013; 10.3928/01484834-20180921-08 30277547

[18] Dekeyser Ganz F, Toren O (2014). Israeli nurse practice environment characteristics, retention, and job satisfaction. Israel journal of health policy research.

[19] Coomber B, Louise Barriball K (2007). Impact of job satisfaction components on intent to leave and turnover for hospital-based nurses: a review of the research literature. Int J Nurs Stud.

[20] Turner BS. Medical power and social knowledge. London: Sage; 1995.

[21] Upreti P (2008). A study to assess the level of job satisfaction among nursing personnel working at BPKIHS..(unpublished dissertation). BP Koirala institute of health sciences.

[22] Ayamolowo SJ, Irinoye O, Oladoyin MA (2013). Job satisfaction and work environment of primary health care nurses in Ekiti state, Nigeria: an exploratory study. Int J Caring Sci.

[23] Hipwell AE, Tyler PA, Wilson CM (2011). Sources of stress and dissatisfaction among nurses in four hospital environments. Br J Med Psychol.

[24] Negussie N. Relationship between rewards and nurses’ work motivation in Addis Ababa hospitals. Ethiopian journal of health sciences. 2012;22(2) PMC3407833 22876074

[25] Mohite N, Shinde M, Gulavani A (2014). Job satisfaction among nurses working at selected tertiary care hospitals. International Journal of Science and Research (IJSR).

[26] ILO, ICN, WHO, & PSI. Workplace Violence in the Health Sector Country Case Study – Questionnaire. Geneva: WHO; 2003.

[27] AbuAlRub RF, Al-Asmar AH (2014). Psychological violence in the workplace among Jordanian hospital nurses. J Transcult Nurs.

[28] Albashtawy M (2013). Workplace violence against nurses in emergency departments in Jordan. Int Nurs Rev.

[29] Hanson GC (2015). Workplace violence against homecare workers and its relationship with workers health outcomes: a cross-sectional study. BMC Public Health.

[30] Hinchberger PA (2009). Violence against female student nurses in the workplace. Nurs Forum.

[31] Lepping P (2013). Percentage prevalence of patient and visitor violence against staff in high-risk UK medical wards. Clinical medicine.

[32] Boafo IM, Hancock P, Gringart E (2016). Sources, incidence and effects of non-physical workplace violence against nurses in Ghana. Nursing Open.

[33] Miers, M., Gender Issues and Nursing Practice 2000, London: MacMillan.

[34] Boafo IM, Hancock P. Workplace violence against nurses: a cross-sectional descriptive study of Ghanaian nurses. SAGE Open. 2017;7(1)

[35] Franz S (2010). Aggression and violence against health care workers in Germany--a cross sectional retrospective survey. BMC Health Serv Res.

[36] Al-Omari H (2015). Physical and verbal workplace violence against nurses in Jordan. Int Nurs Rev.

[37] Çelik Y, Çelik SŞ (2007). Sexual harassment against nurses in Turkey. J Nurs Scholarsh.

[38] Gacki-Smith J (2009). Violence against nurses working in US emergency departments. J Nurs Adm.

[39] Nachreiner NM (2007). Difference in work-related violence by nurse license type. J Prof Nurs.

[40] Yaktin US, Azoury NB-R, Doumit MA (2003). Personal characteristics and job satisfaction among nurses in Lebanon. J Nurs Adm.

[41] Addae S. The evolution of modern medicine in a developing country: Ghana 1880–1960. Durham: Academic Press; 1996.

[42] Laschinger HKS (2004). Hospital nurses’ perceptions of respect and organizational justice. J Nurs Adm.

[43] Kangas S, Kee CC, McKee-Waddle R (1999). Organizational factors, nurses’ job satisfaction, and patient satisfaction with nursing care. J Nurs Adm.

[44] Barrett L, Yates P (2002). Oncology/haematology nurses: a study of job satisfaction, burnout, and intention to leave the specialty. Aust Health Rev.

[45] Larrabee JH (2003). Predicting registered nurse job satisfaction and intent to leave. J Nurs Adm.

[46] Usmani S, Jamal S (2013). Impact of distributive justice, procedural justice, interactional justice, temporal justice, spatial justice on job satisfaction of banking employees. Review of Integrative Business and Economics Research.

[47] Al-Zu’bi HA (2010). A study of relationship between organizational justice and job satisfaction. International Journal of Business and Management.

[48] Thomas P, Nagalingappa DG (2013). Consequences of perceived organizational justice: an empirical study of white-collar employees. Journal of Arts, Science & Commerce.

[49] Cotter DA, Hermsen JM, Vanneman R (2003). The effects of occupational gender segregation across race. Sociol Q.

[50] Purcell D, MacArthur KR, Samblanet S (2010). Gender and the glass ceiling at work. Sociol Compass.

[51] Rosenstein AH (2002). Nurse-physician relationships: impact on nurse satisfaction and retention. AJN The American Journal of Nursing.

[52] Sirota T (2008). Nurse/physician relationships survey report. Nursing.

[53] Gamarnikow E, Kuhn A, Wolpe A (1978). Sexual division of labour: the case of nursing. Feminism and materialism: women and modes of production.

[54] Ghana Statistical Service (GSS) (2012). 2010 Population & Housing Census: summary report of final results.

[55] Leape LL (2012). Perspective: a culture of respect, part 1: the nature and causes of disrespectful behavior by physicians. Acad Med.

[56] Ezzat HA, Lashin O (2005). Violence on hospital nurses and job satisfaction. The new Egyptian Journal of Medicine.

[57] Pillinger J (2011). Quality health care and workers on the move: Ghana National Report.

[58] WHO (2016). Global strategy on human resources for health: workforce 2030.

[59] Greenberg J (2001). Setting the justice agenda: seven unanswered questions about “what, why, and how”. J Vocat Behav.

[60] Nagy MS (2002). Using a single-item approach to measure facet job satisfaction. J Occup Organ Psychol.

[61] Siegrist J (1996). Adverse health effects of high-effort/low-reward conditions. J Occup Health Psychol.

[62] Mrayyan MT (2007). Jordanian nurses’ job satisfaction and intent to stay: comparing teaching and non-teaching hospitals. J Prof Nurs.

[63] Boafo IM, Hancock P, Gringart E (2016). Sources, incidence and effects of non-physical workplace violence against nurses in Ghana.

[64] Pallant J (2013). SPSS survival manual.

[65] Mrayyan MT (2006). Jordanian nurses’ job satisfaction, patients’ satisfaction and quality of nursing care. Int Nurs Rev.

[66] Budin WC (2013). Verbal abuse from nurse colleagues and work environment of early career registered nurses. J Nurs Scholarsh.

[67] Gerberich SG (2004). An epidemiological study of the magnitude and consequences of work related violence: the Minnesota nurses’ study. Occupational & Environmental Medicine.

[68] Harris B, Leather P. Levels and consequences of exposure to service user violence: evidence from a sample of UK social care staff. Br J Soc Work. 2011:1–19.

[69] Purpora C, Blegen MA (2015). Job satisfaction and horizontal violence in hospital staff registered nurses: the mediating role of peer relationships. J Clin Nurs.

[70] Al-Dossary R, Vail J, Macfarlane F (2012). Job satisfaction of nurses in a Saudi Arabian university teaching hospital: a cross-sectional study. Int Nurs Rev.

[71] Ham J (2013). The relation among nurses’ experience of verbal abuse, social support and their turnover intention.

[72] Sarpong PA. Ghana in retrospect. Tema: Ghana Publishing Corporation; 1974.

